# Automated Engineering Protein Dynamics via Loop Grafting: Improving *Renilla* Luciferase Catalysis

**DOI:** 10.1021/acscatal.4c06207

**Published:** 2025-02-11

**Authors:** Joan Planas-Iglesias, Marika Majerova, Daniel Pluskal, Michal Vasina, Jiri Damborsky, Zbynek Prokop, Martin Marek, David Bednar

**Affiliations:** † Loschmidt Laboratories, Department of Experimental Biology and RECETOX, Faculty of Science, Masaryk University, Kotlarska 2, Brno 602 00, Czech Republic; ‡ International Clinical Research Centre, St. Anne’s University Hospital, Pekarska 53, Brno 602 00, Czech Republic

**Keywords:** loop grafting, protein dynamics, bifunctional protein chimeras, Renilla luciferase, enzyme engineering, bioluminescent catalytic activity

## Abstract

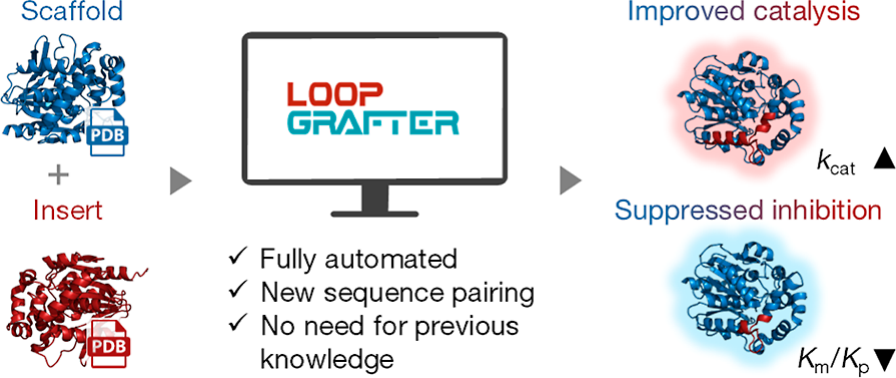

Engineering protein dynamics is a challenging and unsolved problem in protein design. Loop transplantation or loop grafting has been previously employed to transfer dynamic properties between proteins. We recently released a LoopGrafter Web server to execute the loop grafting task, employing eight computational tools and one database. The LoopGrafter method relies on the prediction of the local dynamic behavior of the elements to be transplanted and has successfully reconstructed previously engineered sequences. However, it was unclear whether catalytically competitive previously uncharacterized designs could be obtained by this method. Here, we address this question, showing how LoopGrafter generates viable loop-grafted chimeras of luciferases, how these chimeras encompass the activity of interest and unique kinetic properties, and how all this process is done fully automatically and agnostic of any previous knowledge. All constructed designs proved to be catalytically active, and the most active one improved the activity of the template enzyme by 4 orders of magnitude. The computational details and parameter optimization of the sequence pairing step of the LoopGrafter workflow are revealed. The optimized and experimentally validated loop grafting workflow available as a fully automated Web server represents a powerful approach for engineering catalytically efficient enzymes by modification of protein dynamics.

## Introduction

A wide range of industrial- and research-oriented applications strive to find more efficient or specific catalysts to perform certain chemical transformations. Enzyme-assisted formation of new bond classes such as Si–C,^1^ the formation of new chiral building blocks relevant to different therapeutical applications,^2^ or the optimization of a bioluminescent luciferase for a more efficient and stable visual reporting^3^ are successful outcomes of such endeavors. Over the last few decades, technological and methodological improvements have led to significant breakthroughs in the understanding and control of catalytic steps, overcoming reaction bottlenecks, and the enhancement of specific production yields. In parallel and blind to human efforts, over the course of billions of years by the action of natural selection and evolution, nature has refined natural biocatalysts, enzymes, to high levels of activity and specificity. It is unsurprising, thus, that when natural enzymes are suitable to a particular chemical task, these are often taken as a starting point for protein engineering efforts, aiming to improve specific properties of the target enzyme by modifying its amino-acidic sequence^4−7^·

Human-driven efforts to improve the properties of natural enzymes rely on two complementary strategies: laboratory experimentation and *in silico* predictions. Among the first, a strategy mimicking natural selection by iterating over rounds of random introduction of mutations on a starting enzyme template and then screening for the desired property improvement has gained traction: directed evolution.^8^ The technique was pioneered by Frances Arnold^9,10^ and was awarded the Nobel Prize in Chemistry in the year 2018.^11^ While on its inception, directed evolution relies on point substitutions, other modifications such as insertion and deletions (indels) play a major role in driving the evolution of proteins;^12^ indels allow more drastic structural and functional changes^13^ and have been on the goal list of protein engineers since for a long time.^14^ Several experimental methods aimed to introduce indels in a template protein randomly have emerged during the last two decades.^15−17^ Following the rationale of introducing larger modifications for a more substantial effect, chimeric design proteins have been posited^18^ and proven^19^ an apt technique to produce improved enzymes. In short, chimeras are generated by replacing parts of a template enzyme with others from a more convenient one.

Among the protein regions targeted for chimerogenesis, loops, the aperiodic regions connecting more steady regular secondary structures, outstand.^20^ Pioneered by Kim and collaborators,^21^ the initial experiments transplanting a loop from two related proteins required multiple rounds of directed evolution (each involving libraries of tens of millions of different mutants). The final evolved enzymes improved their activity around 160 times, after undertaking a large number of mutations around the transplantation recombination points;^21^ thus, the final products were not pure chimeras but evolved ones. Loops have become popular targets of transplantation or grafting because their flexible properties are often related to protein thermostability,^22^ function,^23,24^ or allostery,^25^ catalytic activity, and substrate specificity.^26,27^ Multiple loop grafting efforts have been carried out recently, improving the target enzyme enantioselectivity,^28^ varying its catalytic efficiency,^29^ improving activity,^30^ or changing substrate specificity.^31^ Noteworthily, most of these efforts required further mutagenesis along the recombination regions or trying multiple possible recombination points. Our group also recently contributed to the field by increasing the bioluminescent activity and modulating the dynamics of a bifunctional luciferase (Luc)/haloalkane dehalogenase (HLD) ancestor^32^ by loop grafting.^33^

On the other hand, a scarcity of *in silico* methods for predicting the effects of dynamical loops on the activity of an enzyme has been noted.^34^ Most of the existing efforts have been focused on loop remodelling,^35^ and only a handful can be repurposed for loop grafting: Loop Modeling,^35^ DaReUs Loop,^36^ and FALC.^37^ The methods often require the final chimeric protein sequence as input and are generally sensitive to the selection of the so-called “anchoring points”, the sequence recombination spots. This is rather unsurprising since a retrospective study showed that successful loop design by transplantation requires a precise structural overlay of the source and the target structures around the transplanted region.^38^ Based on this observation, we recently developed a final-sequence-agnostic Web-based tool, LoopGrafter,^39^ to aid the loop transplantation effort from one insert protein to a homologous scaffold template.

Here, we reveal the details of the computational workflow and optimized parameters of LoopGrafter, specifically on the step that allows establishing potential recombination points between the source and target proteins. We also experimentally validate the loop grafting strategy to fully automate the loop transplantation process and produce novel viable and catalytically competitive chimeras. Our methodology relies on exploring both the local flexibility and the recombination point spaces of the proteins involved in the transplant. The first allows targeting regions likely involved in similar dynamic processes; the second enables identifying optimal residue spots for the transplant and has parallels to recent methods applied to chimeric domains.^40^ Using this methodology, we present here eight new chimeric loop-grafted Luc/HLD variants, different to those previously reported,^33,41,42^ showing that all of them increase the bioluminescence levels and exhibit different predicted flexibility patterns when compared to the template protein.^33^ We further kinetically characterize three of them, showing that they explore the trade-off between substrate affinity and catalytic rate. These newly designed biocatalysts provide direct experimental evidence for the feasibility of our fully automated methodology as a useful tool to explore the protein dynamics space in order to increase its enzymatic activity.

## Methods

### Alignment of Input Proteins

The crystallographic three-dimensional (3D) structures of the wild type variant of the bifunctional ancestor (PDB ID: 6g75,^32^ chain A) and the closed conformation of *Renilla* Luciferase 8 (PDB ID: 2psf,^43^ chain B) were downloaded from RCSB PDB^44^ and superimposed using combinatorial extension (CE).^45^ This superimposition was used as input to an efficient greedy algorithm that aligns the closest alpha carbons among the two structures (see Supporting Information), given that the pair is not further away than 1.9 Å and its sequence offset does not differ more than 4 times the standard deviation of the whole superimposition distribution (adjustable parameters). The output of the algorithm is a structure-based sequence alignment between the two input proteins that can be further used to inform the possible recombination points between the two. For comparison purposes, we also used a new algorithm on the alignment of a previously reported chimeric Luc/HLD variant (AncFT7, PDB ID: 7ome)^42^ to 6g75 and 2psf. We performed the same alignments using a novel sequence-based alignment method (PLMSearch,^46^ using default parameters on the Web server) and a recent structure-based alignment algorithm (GTalign,^47^ installed from git repository and using default parameters).

#### Secondary Structure Determination

The secondary structure for each amino acid in the scaffold and insert proteins was determined by DSSP^48^ from their 3D coordinates and then manually adjusted upon visual inspection on PyMOL.^49^ The final assignment strictly followed the definitions we used previously in the original transplantation effort on the bifunctional Luc/HLD.^33^

### Flexibility Evaluation and Cross-Correlation

Flexibility on the input scaffold protein was calculated using two variants of elastic network models (ENMs), Gaussian (GNM)^50,51^ and Anisotropic (ANM),^52^ as implemented in ProDy.^53^ The number of modes was set to the amino-acid sequence length of the input scaffold protein (bifunctional HLD/LUC ancestor^32^) minus one. The calculation of cross-correlations and square fluctuations was required from the program. The outputs obtained were (i) the average flexibility values per α carbon (in the form of B factors), (ii) the average square fluctuations ((Δ*R*_i_)^2^) for each α carbon i from which the individual B factors (*B*_i_) are calculated
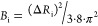
1and (iii) the cross-correlations of square fluctuations in between any pair of residues. The squared fluctuations were further used to calculate average *B* values per region. Secondary structure elements (regular helices and strands and aperiodic coils) and supersecondary structures (an aperiodic coil and its flanking regular structure elements) were assigned average flexibility and flexibility cross-correlation values as described before.^33^

### Determination of Boundaries of the Transplanted Region

The sequence pairing in the loops region was used to guide the decision about the boundaries of the insert design (see above and Supporting Information). For each supersecondary structure or loop region (comprised of the coil segment and its flanking regular secondary structure elements, SSEs), each position on the obtained sequence pairing represents a potential boundary for the insert (a recombination point). The boundary is represented by the pair of residues (one from the insert and one from the scaffold) that define the initial or ending position of the transplant. However, some of the positions might not provide sequence novelty, and gapped positions offer multiple choices for defining the boundary. To solve these problems, the boundaries were iteratively explored at each position on each of the SSEs, starting from the residue farther away (most distal) from the coil segment. In this iterative process, whenever there was no gap, a boundary position on the scaffold and on the insert was defined by the two paired amino acids. If there was a gap present in the sequence pairing, the unaligned residue was paired with the gap-flanking residue (anterior or posterior) at the shortest Euclidian distance.

This procedure provided two sets of possible boundaries: one for the N-terminal SSE and the other for the C-terminal one. All possible combinations of boundaries, one from the N-terminal SSE, and one from the C-terminal SSE, were considered to compile the design lists. A particular combination was accepted for further evaluation only if the grafted variant provided a sequential variation on the scaffold or any other accepted design.

Partial structures of both the excised scaffold and the insert were generated for each design to be used as input for the generation of the 3D models of the grafted designs. In the case of the scaffold, only one structure was generated with as many regions excised as needed (one or more depending on the number of loops to be grafted). Conversely, one partial structure was produced for each inserted region.

### Generation of Combinations of Transplanted Loops

All possible designs for an individual loop were combined with any other possible designs from a different loop to create loop combination designs. In the present case, only loops 9 and 14 were considered (see Results); thus, designs for loop 9 only, loop 14 only, and the combination of loop 9 and loop 14 were produced.

### Generation of 3D Models of the Designed Grafted Chimeras

MODELLER^54^ was used to generate the 3D model of each of the designed grafts. The accuracy of the results produced by this software is highly dependent on the precision of the sequence alignment. In this case, the alignment provided was based on the one derived from structural superimposition (see above). The alignment for the grafted sequence (also required as input) was derived from the corresponding reference sequence (scaffold for scaffold regions and insert for insert regions).

The default automodel.py script provided with the MODELLER distribution was used to generate the grafted 3D model. Partial structures of the insert and excised scaffold (see Determination of Boundaries of the Transplanted Region above) and the alignment herein described were used as input. As output, MODELLER provides a 3D model of the chimeric grafted design and a Discrete Optimized Protein Energy (DOPE^55^) value to evaluate the goodness of the model (the lower score is the better).

### Evaluation of the Models

Two different scores were used to evaluate the obtained chimeric models. First, DOPE,^55^ as calculated by MODELLER on the produced model. Second, Rosetta combined the score after minimizing the obtained chimeric model with Rosetta FastRelax (FR) using the Talaris 2014 scoring function.^56^ On the available crystals of the chimeric proteins (previously designed bifunctional ancestors^33,42^), we obtained AlphaFold2 models using ColabFold^57^ (we considered only the best ranking model for each ColabFold run), ESMFold^58^ models from online implementation (https://esmatlas.com/resources?action=fold), using default parameters, RoseTTAFold^59^ models from the online tool (https://robetta.bakerlab.org/, all 5 produced models were considered), and SwissModel,^60^ also from the publicly available Web server (https://swissmodel.expasy.org/) using the two different biounits from each 6g75 and 2psf; the best results in terms of (lower) RSMD were obtained using 6g75 chain A as a template. In all cases, default parameters were used. The obtained models were superimposed on the crystallographic structures using a CE. By means of this superimposition, the RMSD over all alpha carbons between the crystal structure and the models was obtained for comparison purposes.

### Predicting Flexibility Properties on the Models

Eight variants (see Results: Selection of Loops to Graft and Pipeline Execution) were selected for further experimental validation, according to their MODELLER DOPE^55^ and Rosetta FastRelax scores.^56^ The flexibility of each the variants along with that of the previous constructs AncFT^33^ and AncFT7^42^ was subsequently predicted using GNM^50,51^ or ANM.^52^ Particularly, B factors derived from predicted root mean squared fluctuations were calculated as described above (see Flexibility Evaluation and Cross-Correlation), setting the number of nodes to the amino-acid sequence length of the analyzed chimeric protein, and the resulting values were normalized (with reference to the maximum and minimum calculated *B*-factor values) for comparison.

### Cell Transformation

*Escherichia coli* BL21(DE3) cells were transformed with 1 μL of expression plasmid vector pET21b containing the corresponding gene (100 ng/μL), plated on LB agar containing 100 μg/mL ampicillin, and then incubated at 37 °C overnight (14–16 h). The cells transformed with pET21b::Anc^HLD-RLuc^^32^ were used as positive controls for the AncFT variants.

### Small-Scale Production and Affinity Purification

Several *E. coli* colonies were streaked to inoculate 2 mL of starting media (2× LB supplemented with 0.5% glucose and 100 μg/mL ampicillin) in a 24-deep-well plate (GE Healthcare, UK). The plate covered with an air-pore membrane was incubated at 37 °C for 4 h, 200 rpm. After incubation, 2 mL of induction media (2× LB supplemented with 0.6% lactose, 50 mM HEPES (pH 7.4), 0.5 mM IPTG, and 100 μg/mL ampicillin) was added. The plate was covered with an air-pore membrane and was incubated at 22 °C for 16 h, 200 rpm. Cells were harvested by centrifugation using a Sigma 6K-15 centrifuge (SciQuip, UK) for 10 min, 1519*g*, and 4 °C, and cells were resuspended in 1.3 mL of a purification buffer (50 mM NaCl, 10 mM imidazole, 10 mM Tris, pH = 7.5). The cells were then disrupted by sonification using Sonic Dismembrator Model Q700S (FisherBrand, USA), and the whole soluble fraction was clarified by centrifugation for 20 min, 3572*g*, and 4 °C. For affinity purification, the soluble fraction was added to TALON SuperFlow Metal Affinity Resin (Takara) and incubated for 2 h on a roller (40 rounds/min) at 4 °C. Unbound proteins were washed twice by centrifugation 94*g* for 2 min followed by resuspending in purification buffer. After the second wash, 40 μL of SDS-PAGE loading buffer (2× Laemmli sample buffer with DTT) was added to each protein/resin sample and incubated for 10 min at room temperature. 1 μL of Color Prestained Protein Standard, Broad Range 10–250 kDa (New England Biolabs, USA) and samples were loaded on SDS-PAGE gel. SDS-PAGE conditions: 400 mA, 200 V, 40 min. After staining with InstantBlue (Missouri, USA) for 20 min, the gel was washed with water for 20 min.

### Cell Cultivations for Enzymatic Screenings

Single colonies of transformed cells were transferred into sterile 96-well microtiter plates (MTP) containing 100 μL of LB medium supplemented with ampicillin (100 μg/mL). The plates were covered with an air-pore membrane and cultivated for 3 h at 37 °C and 200 rpm. After cultivation, an additional 100 μL of LB medium with ampicillin (100 μg/mL) and IPTG (1 mM) was added to the minicultures, and the MTP was afterward incubated at 20 °C, 200 rpm for 18 h. The optical density (OD_600_) of MTP cultures was determined spectrophotometrically and cultures were harvested by centrifugation at 4 °C, 1600*g* for 20 min. The pellet was immediately frozen at −70 °C.

### Large-Scale Protein Production and Purification

Several colonies of *E. coli* strain cells BL21(DE3) transformed with pET21b containing the desired gene were streaked to 10 mL of LB media containing ampicillin (100 μg/mL) and cultivated for 4 h at 37 °C. After incubation, the whole volume was poured into 1 L of LB media containing ampicillin (100 μg/mL) to start the main culture and cultivated at 37 °C until the OD_600_ reached 0.6. The enzyme expression (at 20 °C) was induced by adding IPTG to a final concentration of 0.5 mM. The cells were incubated for 16 h at 20 °C and harvested at 4500*g*, 15 min. The pellet was resuspended in 30 mL of purification buffer with a composition of 20 mM phosphate buffer, 400 mM NaCl, 10 mM imidazole, pH = 7.5, and frozen at −70 °C. Prior to the purification, cultures were thawed in lukewarm water, and 90 μL of DNase (1 mg/mL) was added. After sonication (Sonic Dismembrator Model 705 Fisher Scientific, USA) in 6 × 2 min cycles with a 50% amplitude (5 s pulse, 5 s pause), lysates were clarified by centrifugation (21,036*g*, 4 °C, 1 h) using a Sigma 6–16K centrifuge (SciQuip, UK) equipped with a 12,166 rotor. The supernatant was filtered using a 0.45 nm MilliPore filter, and the samples were applied to the HisTrap Excel column and purified using affinity chromatography (KTA Pure, Cytiva, USA). After elution (20 mM phosphate buffer, 400 mM NaCl, 500 mM imidazole, pH = 7.5) and dialysis (50 mM phosphate buffer, pH = 7.5), proteins were purified in the second step by size exclusion chromatography (KTA Pure, Cytiva, USA) equipped with a HiLoad 16/600 Superdex 75 pg column. The protein purity was verified by SDS-PAGE, and the same protocol was used for all of the AncFT variants.

### Thermostability Measurement

The thermostability of purified enzymes was measured on nanoDSF (differential scanning fluorimetry, Prometheus NT.48, NanoTemper Technologies, GmbH), in the temperature range from 20 to 95 °C and at a rate of 1 °C/min. The measurement was performed in three independent experiments with a concentration of samples around 1 mg/mL. The melting temperatures (*T*_m_) were evaluated directly by ThermControl v2.0.2. And averages together with standard deviations of the data were calculated.

### Luminescence Assays

For the whole-cell extracts screening assay, the MTP cultures were thawed at laboratory temperature for 10 min, and 70 μL of a lysis buffer (20 mM potassium phosphate, 20 mM Na_2_SO_4_, and 1 mM EDTA, pH = 8.0) containing lysozyme (1 mg/mL) was added to each well. The MTP was incubated at 23 °C, for 1 h, and cell debris was removed from the lysate by centrifugation at 1600*g* for 20 min. The supernatant was transferred into a new MTP, and the pellet was discarded. Cells transformed with an empty vector were used as a negative control, and luminescence of AncFT was measured as positive control. For the determination of purified enzymes luminescence, the concentration of enzymes was determined spectrophotometrically (DS-11 Spectrophotometer, DeNovix, USA). The determined concentrations were 5.2 mg/mL for AncFT9, 7.2 mg/mL for AncFT15, and 4.7 mg/mL for AncFT16. The enzyme samples for luciferase activity measurements were diluted by a factor of 200. The 25 μL aliquots of cell-free extract or purified enzymes were transferred into a new MTP, and the baseline luminescence signal was measured for 5 s, gain 2200, using a FLUOstar Omega microplate reader (BMG Labtech, Germany). A 225 μL aliquot of assay buffer (100 mM potassium phosphate, 1 mM Na_2_SO_4_, pH = 7.5) supplemented with 8.8 μM coelenterazine was added to cell lysates/protein samples, and luminescence was measured immediately at 37 °C, for 68 s, with gain 2200. In order to compare the luciferase activity of the cell screening assay, the results of all variants were normalized to a cell OD_600_ value of 1. All data for the luminescence assay were measured in triplicate. All data were measured in triplicate. The relative activity was calculated from the peak area, and averages with standard deviations were calculated from the three measurements. The relative activity in the cell screening assay was calculated in RLU·s^–1^, where RLU represents relative luminescence units. For purified enzymes, the relative activity was calculated as RLU·s^–1^·mg^–1^. Average activity with a standard deviation of three measurements was determined for all variants, and fold increase in activity of all variants was calculated toward the Anc^HLD-RLuc^.

### Analysis of Enzyme Steady-State Kinetics

Steady-state kinetic parameters of the luciferase reaction were determined at 37 °C by using the FLUOstar Omega microplate reader (BMG Labtech, Germany). A series of buffered coelenterazine solutions (4.4 × 0.5^0–7^ μM) were prepared by dilution of ethanolic coelenterazine stock solution (∼650 μM) into 100 mM potassium phosphate buffer (pH 7.50). 25 μL of purified enzyme solution was placed in a well of a white opaque microtiter plate (OptiPlate-96, PerkinElmer, USA), and after 10 s baseline collection, the luciferase reaction was initiated by injection of 225 μL of buffered coelenterazine solution. Total luminescence (240–740 nm) was monitored for 1000 s or until the luminescence intensity decreased under 0.5% of its maximal measured value, i.e., until the substrate was fully converted to product; this was performed for the entirety of the coelenterazine concentration series. The final enzyme concentration was either 0.1 or 0.01 μM. Each measurement was performed in at least three repetitions.

**Figure 1:**
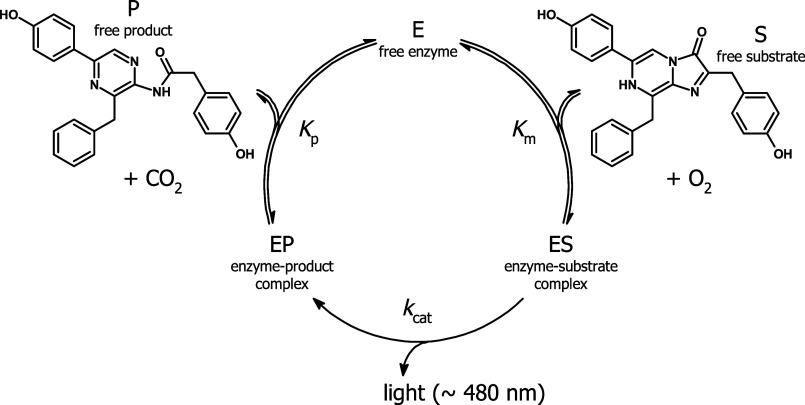
Steady-state kinetic model for the luciferase reaction optimized by the loop grafting methodology.

Unlike the classical initial velocity analysis, we have applied an updated protocol for collecting and fitting steady-state luminescence kinetic data.^33^ Monitoring the luciferase reaction beyond the initial linear phase up to the complete conversion of the substrate allows for precise scaling of the luminescence to product concentration without the need for complicated quantum yield calibration. The recorded traces of dependences of luminescence intensity on the reaction time, which are proportional to d([EP] + [P])/d*t*, were transformed into cumulative luminescence over time, which is proportional to ([EP]+[P])/*t*. The obtained transformed kinetic data were globally fitted by numerical integration methods using the KinTek Explorer^61^ (KinTek Corporation, USA). By numerically integrating the rate equations automatically derived by the software from the input kinetic model (Figure 1) and incorporating the function that scales luminescence to product concentration, we were able to directly determine the values of turnover number *k*_cat_, Michaelis constant *K*_m_, specificity constant *k*_cat_/*K*_m_, and equilibrium dissociation constant for enzyme product complex *K*_p_. To reflect fluctuation in experimental data, the values of substrate or enzyme concentrations were corrected (±5%) to obtain the best fits. Residuals were normalized by the sigma value for each data point. The standard errors (SE) were calculated from the covariance matrix during nonlinear regression. In addition to conventional estimates of SE values, a more rigorous variation of the kinetic parameters was performed by confidence contour analysis using FitSpace Explorer^62^ (KinTek Corporation, USA). In this analysis, the lower and upper limits for each parameter were derived from the confidence contours for the χ^2^ threshold at boundary 0.9. Depletion of the available substrate after the reaction was verified by repeated injection of a fresh enzyme, resulting in no or negligible luminescence.

## Results

### Computational Workflow of LoopGrafter

**Figure 2:**
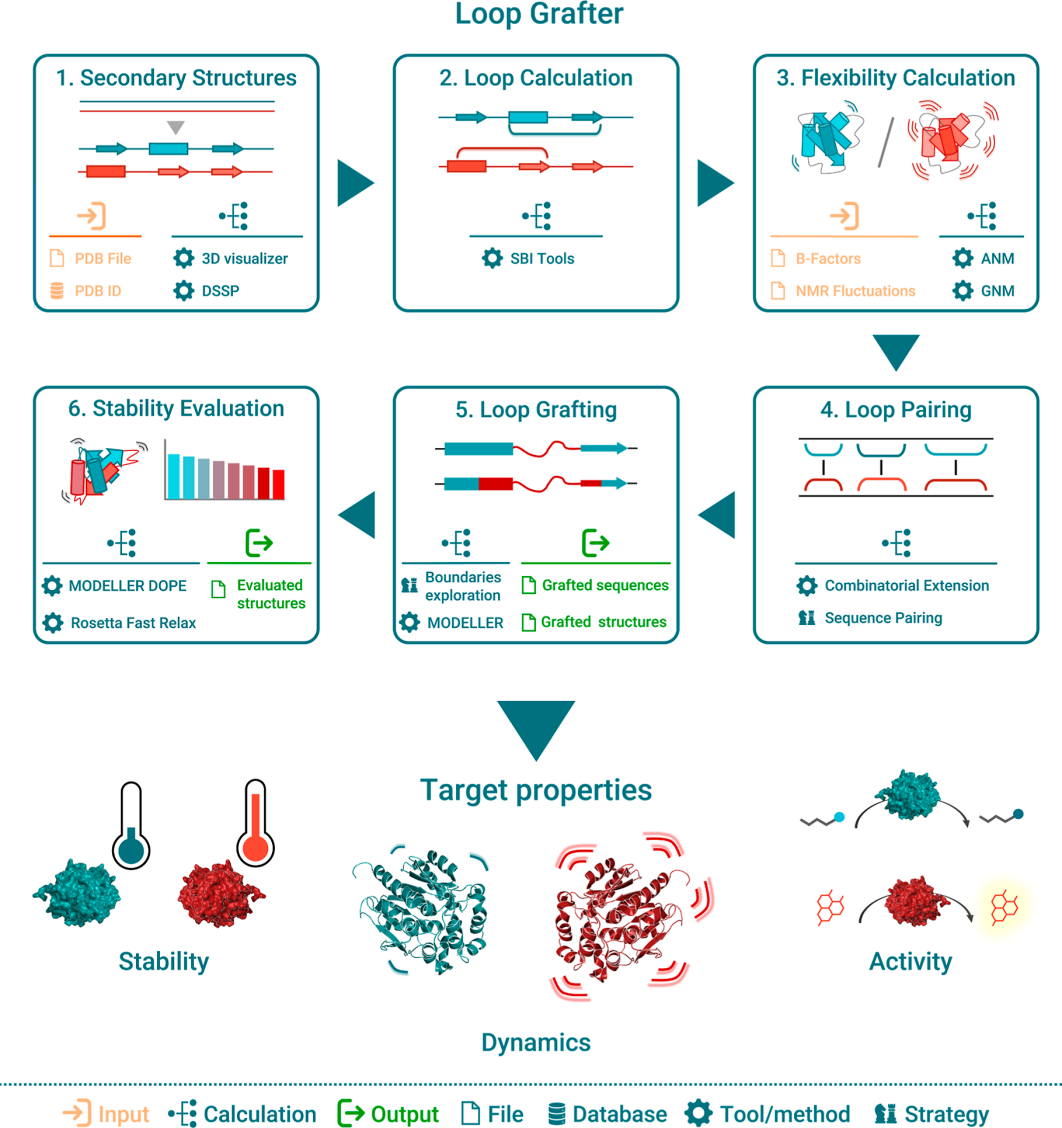
LoopGrafter workflow scheme in the context of transplanting a dynamic loop from *Renilla* luciferase to a hyperstable haloalkane dehalogenase template. Different sequential procedures (edges, clockwise) lead to successful loop grafting (center) exemplified in the transplant of a loop from *Renilla* luciferase to a hyperstable haloalkane dehalogenase template.

LoopGrafter is an automated pipeline to transplant loops between homologous proteins divided in six logical steps: (i) secondary structure definition; (ii) loop exploration; (iii) flexibility evaluation; (iv) loop pairing; (v) recombination points definition (loop grafting); and (vi) stability evaluation (Figure 2). After selecting the structures of the protein with one or more loops to replace (scaffold) and the protein whose loops will be transplanted (insert), SSEs are calculated with DSSP (and may be adjusted manually) and the superimposition of the two proteins can be visualized, i.e., using PyMOL^49^ (Figure 2, **panel 1**). SBILib^63^ is used to calculate loops on the input structures (Figure 2, **panel 2**). Flexibility in the form of predicted B factors and cross-correlations is calculated using Anisotropic^52^ and Gaussian^50^ network models (ANM and GNM, respectively; Figure 2, **panel 3**). Custom-introduced values can be considered at this step. To identify which loops should be exchanged and if they are compatible, the structures of the two input sequences are superimposed using CE,^45^ and a sequence alignment is derived from such structural superimposition (Figure 2, **panel 4**). This step is key to inform of possible recombination points and is explained in detail below. LoopGrafting (Figure 2, **panel 5**) consists of iteratively exploring all possible recombination points between scaffold and target proteins as given per the previously derived sequence alignment. The procedure, which has been described before,^39^ generates multiple chimeric sequences and its outputs are required by MODELLER to reconstruct the 3D coordinates of each putative chimera. Finally, the stability of each generated 3D structure is evaluated by MODELLER DOPE and Rosetta FastRelax (FR) score (Figure 2, **panel 6**). Our loop grafting strategy targets flexible regions in proteins, assesses the stability of the constructed chimeras, and potentially targets activity aspects of the resulting chimeric enzyme (Figure 2, bottom).

### Structure-Derived Sequence Pairing Procedure and Parameters Optimization

A key step in the loop grafting procedure is deciding the recombination points where the material from the two proteins involved in the transplant will be exchanged. To engage with this task, we take into account the following considerations. First, in crystallographic energy minima, protein loops tend to adopt a limited number of conformations.^64^ In contrast, conformational changes in the coil region of loops are frequently observed to drive enzyme function or regulation.^65^ Thus, selecting recombination points in the flanking, more rigid regular secondary structures becomes a logical choice. The next issue to solve is that recombination points must be chosen on both of the participating proteins at the same time. To address this problem, the operation is often done over a sequence alignment.^31,66−68^ However, our approach relies on geometrical similarity between the regions to be transplanted. To solve this conundrum, mapping between structural superimposition and sequence alignment should be obtained. Some of the most widely used methods for structural superimposition (i.e., super and align as implemented in PyMOL) minimize the pairwise residue–residue distances based on a precalculated sequence alignment. Although these methods produce a handy sequence alignment, they are not useful in our approach because the 3D structural superimposition is driven by sequence and not by structure. An alternative is to use methods that produce sequence alignment from structural superimposition. *T*_m_-align^69^ achieves the feat calculating a global optimum of pairwise residue–residue distances, at the price of potentially mismatching close residues for the benefit of the global alignment. FATCAT^70^ alternatively assumes local flexibility and aligns protein segments by similar proximity; as a result, it may produce a discontinuous protein superimposition. Since none of the available solutions is perfectly suited to our problem, we decided to use CE^45^ for the superimposition of proteins, which is driven exclusively by geometrical similarity. From this superimposition, a matrix *D* containing all pairwise Euclidian distances between the alpha carbons of the residues of each of the superimposed proteins can be obtained. In this work, we developed a naïve algorithm that greedily pairs the closest residues in the superimposed structures given that their Euclidian distance is not larger than 1.9 Å (*T*). The natural algorithm was optimized by comparing ranks instead of distances to minimize its execution time. The greedy behavior is desired to enforce the most local structural similarity. Sequence noncorrelativeness is solved by unpairing the noncorrelative matched residues. To avoid aligning residue pairs that are far away in sequence, further unpairing is applied to those pairs with a difference in sequence position 4 times higher than the standard deviation of the distribution (*s*_*ij*_). *T* and *s*_*ij*_ are the only two parameters of the algorithm (apart from the distance matrix *D* describing the input superimposition of 3D coordinates) and have been optimized in a set of approximately 200 haloalkane dehalogenase structures obtained from the PDB. Details on the algorithm and its optimization are provided in Supporting Information. A comparison to state-of-the-art protein sequence alignment methods^46,47^ is provided in Supporting Information (File S1). LoopGrafter alignment closely resembles that of the structure-based GTalign.^47^ As a result, from a geometrical guided structural superimposition, we obtain a sequence alignment that in turn can be used to decide on recombination points for loop transplant. More precisely, all aligned positions in the flanking regular secondary structures become putative recombination points in our workflow.

### Selection of Loops to Graft and Pipeline Execution

Critical experimental validation is an essential component of the development cycle for software tools in the protein engineering domain since only accurate and robust predictions are valuable to experimentalists. In order to validate our approach, we designed two different experiments. First, we tested the ability of LoopGrafter to reproduce the existing structures of *Renilla* luciferase/haloalkane dehalogenase chimeric sequences (PDB IDs: 6s97 for AncFT1 and 7ome for AncFT7).^33,42^ Second, we tested the ability of LoopGrafter to predict the sequence of engineered chimeras encoding loops from a highly active luciferase capable of producing viable, expressible proteins that have interesting catalytic properties. The rationale of this effort is driven by former work on *Renilla* luciferase variants, which showed that different opening/closing regimes of the cap domain for larger/smaller substrates require dynamics alterations.^41^

Existing *Renilla* luciferase/haloalkane dehalogenase chimeric structures had loop 9 (L9 for short, see Figure S1) and loops 9 and 14 (L9, L14) transplanted from *Renilla* luciferase (PDB ID 2psf, chain B) into a hyperstable haloalkane dehalogenase template (PDB ID 6g75, chain A, hereinafter referred to as scaffold protein or Anc^HLD-RLuc^).^32^ Regarding L9, consistently with previous definitions,^33^ we ignored the small helical element in residues 151–153, considering them as coil. Second, we blindly took the best candidates produced by LoopGrafter after transplanting one or two of these loops and analyzed their catalytic properties.

**Figure 3:**
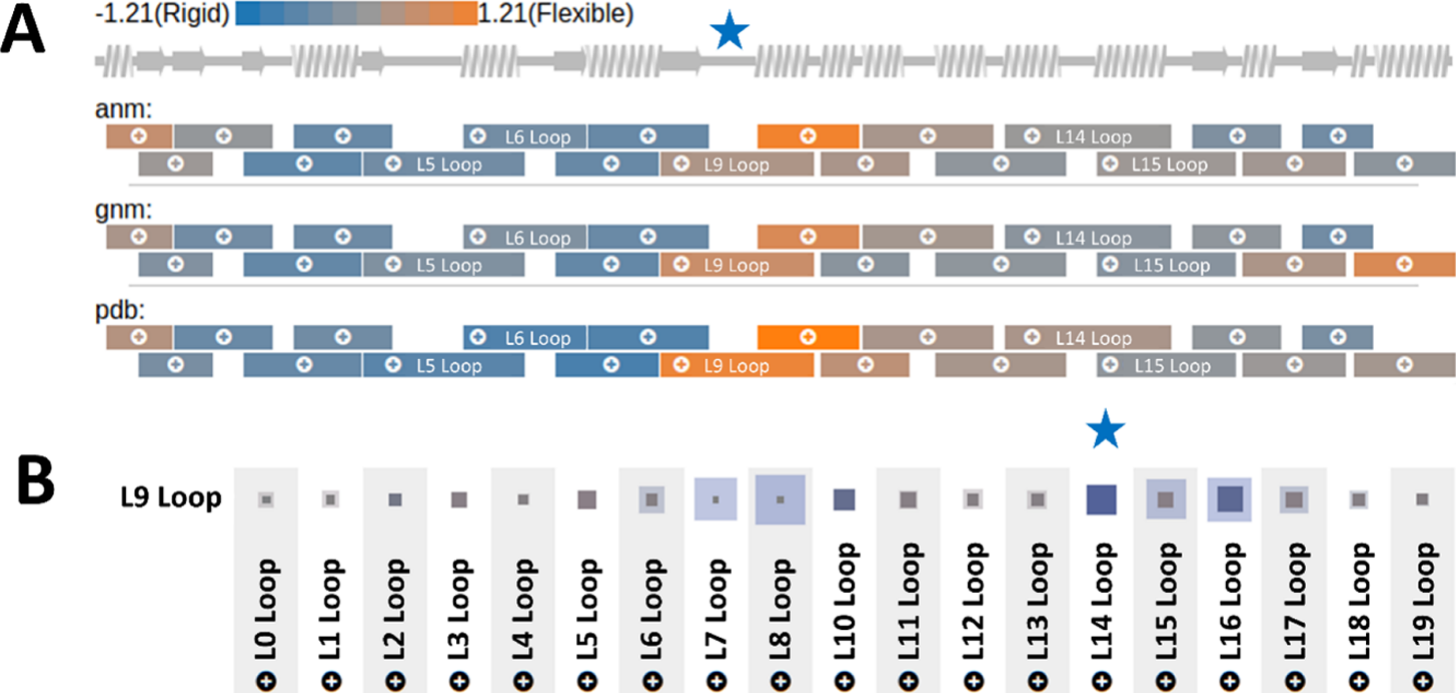
Assessment of scaffold protein flexibility. (A) B factors obtained from the crystallographic record (pdb) or calculated by ENMs (ANM and GNM) mapped to the secondary structure representation of Anc^HLD-RLuc^. The L9 loop (indicated with a star) stands as a dynamic hinge in between the core domain of the protein (N-terminal, rigid, blue color) and the cap domain (C-terminal, flexible, orange color). Note that different loops overlap as the C-terminal regular SSE of loop *n* is the same as the N-terminal regular SSE of loop *n +* 1, thus causing an overlap on their representation. (B) Motion cross-correlation of L9 and other loops in the protein. Semitransparent shade squares correspond to the motion cross-correlation between supersecondary structures (loops defined as two flanking SSEs and a coil section between them). Solid share squares correspond to the motion cross-correlation between the coil part of L9 and any other SSE in any other loop. Blue color corresponds to direct cross-correlation, while red color corresponds to inverse cross-correlation. The L14 loop (indicated with a star) shows strong positive cross-correlation with the coil section of L9.

The flexibility analysis by ENMs performed by LoopGrafter and crystallographic temperature factors revealed that L9 (residues 135–166) acts as a hinge between the rigid main domain of the protein and its more flexible cap domain (starred in Figure 3A). Here, we will refer as flexibility to the local displacements of the backbone of the protein, while we will use the term dynamics to indicate changes of conformational arrangements communicated through the protein (i.e., between different loops). Our analysis interestingly showed that the coil part of the loop is highly cross-correlated (ANM) with that of loop 14 (starred in Figure 3B) suggesting a dynamic cross-talk communication between the loops. Notably, L14 is the region (spanning between residues 210 and 244 and formed by helix 7, coil 14, and helix 8) that was previously grafted with success.^42^ Including those two regions in our pipeline for recombination points exploration, LoopGrafter yielded 474 possible chimeric designs, 18 containing an insert only in the L9 region, 24 only in the L14 region, and 432 in both.

### Evaluation of the Obtained Chimeric Models

Each of the 472 chimeric designs obtained from the pipeline was characterized by the scores it obtained from MODELLER (DOPE^55^) and Rosetta FastRelax (using Talaris 2014 scoring function^56^). The obtained designs ranged between 1731 and 2439 arbitrary MODELLER DOPE units (arbitrary energy units, AU), and between −205 and 602 FastRelax Rosetta energy units (REUs, Table 1). Interestingly, chimeras containing only an L14 insert achieved better (lower) scores than the other designs. This is unsurprising since the coil part of L14 is shorter compared to that of L9. On the other hand, some of the designs encompassing both transplants obtained relatively good scores (a minimum of 1788 DOPE AUs or −261 FastRelax REUs, Table 1), giving hope for the viability of such designs.

**Table 1:** Extreme Scores of Chimeric Designs^a^

grafted loops	DOPE min [AU]	DOPE max [AU]	FR min [REU]	FR max [REU]
loop L9	1731	1995	–205	39
loop L14	1648	2154	–279	–125
loops L9 + L14	1788	2439	–261	602

a Both MODELLER DOPE and Rosetta FastRelax (FR) scores represent arbitrary energy-related units (AU); in the case of FR, the units are designated REUs. Both scoring systems use lower values to indicate higher stability.

**Figure 4:**
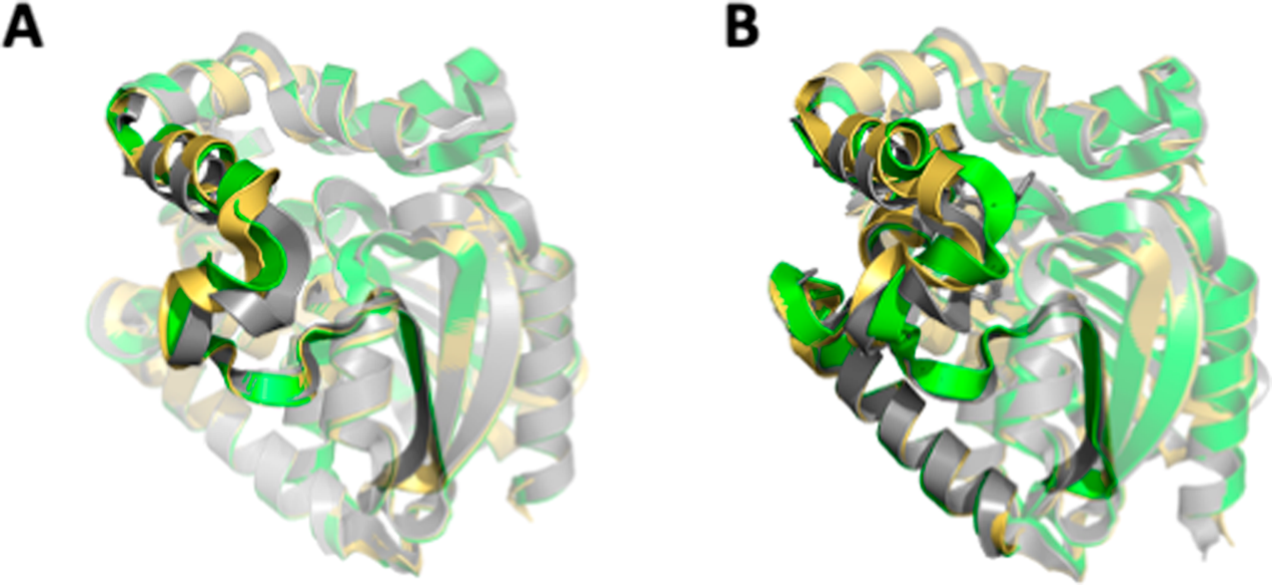
Superimposition of crystal structures and LoopGrafter and AlphaFold models. 3D coordinates of crystal structures (gray), LoopGrafter models (green), and AlphaFold models (yellow) are represented as cartoons for the chimeric proteins including grafted L9 (AncFT1,^33^ A) and grafted L9 + L14 (AncFT7,^42^ B). The grafted portions are highlighted in solid hue; the portions that remained unchanged from the scaffold are shown in semitransparent hue. The alpha-carbon trace along the flexible regions (especially in L9) of the LoopGrafter model is closer to the crystallographic structure than that of the AlphaFold model.

The 3D structure for two of the obtained grafting designs was previously solved by our group using X-ray crystallography.^33,42^ Particularly, one of the structures had only L9 inserted from *Renilla* luciferase 8 (AncFT1) and the other comprised the insert of the two regions L9 and L14 (AncFT7). In order to assess the quality of the 3D models obtained by LoopGrafter, we compared these two crystals with their corresponding models either generated by the pipeline or by third-party homology modeling solutions (AlphaFold,^57^ ESMFold,^58^ RoseTTAFold,^59^ and SwissModel^60^ ). In both cases, the model produced by LoopGrafter conformed better to the crystallographic structure than the model obtained by others, with the exception of SwissModel on the double loop transplant (Figure 4 and Table 2). These results indicate that the fragment-based approach adopted herein is suited to predicting the structure of the resulting grafted proteins.

**Table 2:** Structural Homology between Theoretical Models and Experimental Structures^a^

	AncFT1 (loop L9)	AncFT7 (loops L9 + L14)
LoopGrafter	0.743	0.896
AlphaFold	0.980	1.027
ESMFold	0.980	1.015
RoseTTAFold	1.112	1.156
SwissModel	0.885	0.878

a Crystallographic structures (PDB IDs 6s97 for AncFT1 and 7ome for AncFT7) were superimposed to models obtained either from LoopGrafter,^39^ AlphaFold,^57^ ESMFold,^58^ RoseTTAFold,^59^ and SwissModel,^60^ and the root mean square deviation (RMSD) over all their alpha carbons was calculated. Columns show the RMSD corresponding to the L9-only insert (first) or to the grafted design containing both loops (L9 + L14, second).

### Selection of Variants to Express and Characterize

Among the 474 obtained chimeric designs, we selected a number to proceed with experimental characterization based on their DOPE and FastRelax scores. The two metrics coincided in scoring the lowest (best) two of the designs consisting of the L9-only transplant. Namely, the inserts i1 and i2 (see Table 3) replaced the original sequence, and these two variants (named AncFT8 and AncFT9, respectively) were selected for further validation. From this group, we also chose the third scoring variant in the Rosetta metric (insert i3), naming it AncFT10.

**Table 3:** Summary of Selected Chimeric Variants for Experimental Characterization

variant name	loops grafted	DOPE score [AU]	FastRelax score [REU]	inserts	ins. code
AncFT8	L9	1731	–196	^138^AIVHMESVVDVIESWDEWPDIEEDIALI^166^	i1
AncFT9	L9	1737	–205	^139^IVHMESVVDVIESWDEWPDIEEDI^163^	i2
AncFT10	L9	1800	–177	^139^IVHMESVVDVIESWDEWPDIEEDIALI^166^	i3
AncFT12	L9 + L14	2156	–261	^138^AIVHMESVVDVIESWDEWPDIEEDIALI^166^	i1
				^211^EVRRPTLSWPREIPLVKGGKPDVVQIVRNYNAYL^244^	i6
AncFT13	L9 + L14	1999	–233	^139^IVHMESVVDVIESWDEWPDIEEDIALIK^167^	i4
				^219^WPREIPLVKGGKPDVVQIVR^238^	i7
AncFT14	L9 + L14	1788	–232	^139^IVHMESVVDVIESWDEWPDIEEDIALIK^167^	i4
				^219^WPREIPLVKGGKPDVV^234^	i8
AncFT15	L9 + L14	1811	–145	^138^AIVHMESVVDVIESWDEWPDIEED^162^	i5
				^219^WPREIPLVKGGKPDV^233^	i9
AncFT16	L9 + L14	1958	–164	^138^AIVHMESVVDVIESWDEWPDIEEDIALI^166^	i1
				^219^WPREIPLVKGGKPDVV^234^	i8

A number of designs containing the transplant for loops L9 and L14 were selected as well. Here, we explore two different strategies for scoring the designs. First, the LoopGrafter pipeline was requested to perform the transplant of both loops at once. Second, scoring the transplant of L14 on the best scoring design for L9 according to the DOPE or Rosetta metric (sequential grafting). From the first strategy, the two best Rosetta or DOPE scoring variants were selected for further analysis. The designs denoted with lower REUs consisted of the inserts i1, i6 (AncFT12) and i4, i7 (AncFT13). The designs resulting in lower DOPE units consisted of the inserts i4, i8 (AncFT14) and i5, i9 (AncFT15). It has to be noted that the inserts i4 and i5 replacing the original L9 were not selected in the earlier step. The sequential grafting starting from the best L9-only Rosetta scoring design resulted in AncFT13 (inserts i4 and i7), while starting from the best L9-only DOPE scoring design resulted in a new variant AncFT16, which consisted of the inserts i1 and i8. Table 3 summarizes all the chimeric designs brought forward for further experimental validation.

The proposed chimeric sequences and the previous constructs AncFT^33^ and AncFT7^42^ were evaluated using GNM^50,51^ or ANM^52^ to predict their residue-level flexibility. The predicted flexibility of all the chimeric constructs was notably different to that of the original template Anc^HLD-RLuc^^32^ (see Figure S2). Notably, even those chimeras which did not carry an L14 transplant (AncFT8–10) showed differences in the predicted flexibility along the region of L14 compared to Anc^HLD-RLuc^ (Figure S2B,D), suggesting a modification in their dynamic behavior.

### Characterization of Designed Proteins: Solubility, Stability, and Luminescence

**Figure 5:**
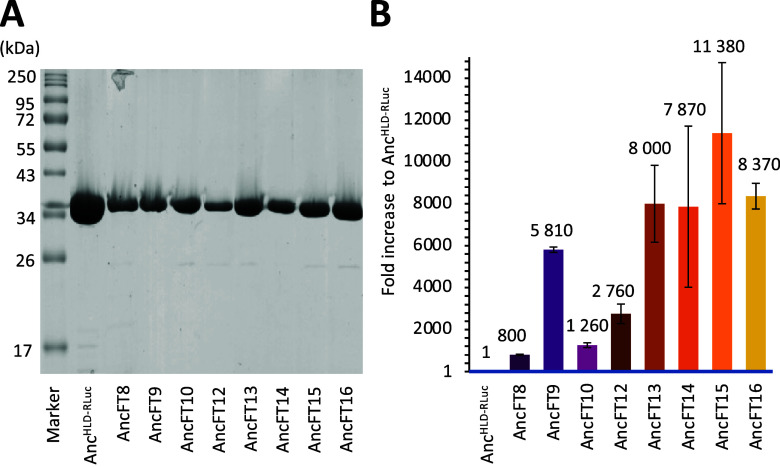
Solubility and luciferase activity screening of new AncFT designs. (A) SDS-PAGE analysis of the solubility of the new chimeric designs. (B) Fold increase of luciferase activity compared to the template Anc^HLD-RLuc^ as determined on soluble whole-cell extracts (in 100 mM potassium phosphate, 1 mM Na_2_SO_4_ buffer assay buffer, pH = 7.5, at 37 °C, for 68 s, with gain 2200). All assays were measured in triplicates (standard deviation depicted as error bars) and normalized to OD_600_ = 1. The baseline activity of the template protein Anc^HLD-RLuc^ (in blue) is arbitrarily set to 1.

To experimentally verify new chimeric designs, all AncFT variants (AncFT8–10 and AncFT12–16) were overproduced in *E. coli* on a small scale. In the first step, His-tagged proteins were affinity-purified, and their solubility was analyzed by SDS-PAGE (Figure 5A). All tested variants were expressed as soluble proteins, as a band corresponding to the expected protein size was detected in all variants. However, while all variants were expressible and soluble, their yields were substantially lower compared to the original template, Anc^HLD-RLuc^ (Figure 5A).

In the second step, proteins were screened for luciferase activity directly in the whole-cell extract using a luminescence assay. Although the amount of overexpressed soluble protein could vary among variants, our results showed that all mutants markedly surpass the luciferase activity of the template bifunctional ancestor Anc^HLD-RLuc^ enzyme (Figure 5B and Table 4). The most promising variants (AncFT9 for L9 grafting and AncFT15 for L9 + L14 grafting) and one representative of sequential grafting (AncFT16) were selected for large-scale purification and more in-depth analysis.

**Table 4:** Summary of Luciferase Activities Determined with the Whole-Cell Extracts of All Loop-Grafted Designs^a^

protein	relative luminescence [RLU s^–1^]	fold increase
AncFT8	6600 ± 230	800 ± 30
AncFT9	47,800 ± 1000	5800 ± 130
AncFT10	103,000 ± 940	1300 ± 120
AncFT12	22,600 ± 3800	2800 ± 470
AncFT13	65,700 ± 15,000	8000 ± 1800
AncFT14	64,600 ± 31,000	7900 ± 3900
AncFT15	93,000 ± 28,000	1100 ± 3400
AncFT16	69,000 ± 5100	8000 ± 600
Anc^HLD-RLuc^	8 ± 0.06	1 ± 0.01

a Relative luminescence activity and standard deviations were calculated from three independent measurements, and the fold increase in activity of all tested variants was compared to that of Anc^HLD-RLuc^. Relative luminescence units (RLU) represent the area of the luminescence peak captured by the FLUOstar Omega microplate reader at buffer, pH, and temperature conditions as in Figure 5 and gain 2200. For comparison, the relative luminescence or RLuc was previously characterized as approximately 250,000 times higher than that of Anc^HLD-RLuc^^32^.

**Figure 6:**
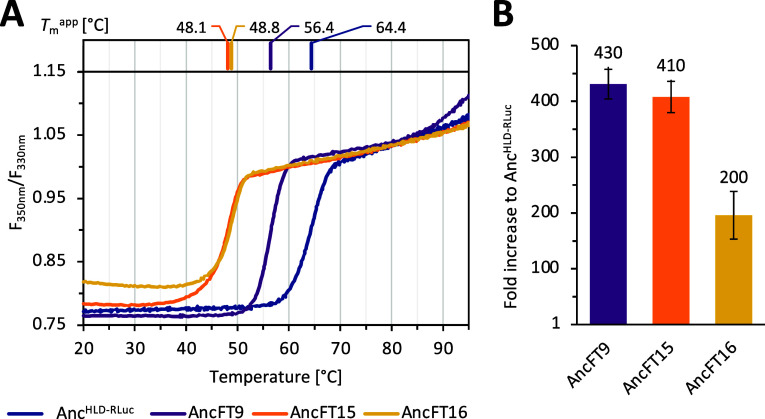
Biochemical characterization of three best loop-grafted designs and template protein Anc^HLD-RLuc^ purified to homogeneity. (A) Thermal unfolding curve (measured from 20 to 95 °C, at a rate of 1 °C/min, see Methods) with a single transition step for each of the variants: Anc^HLD-RLuc^ (blue), AncFT9 (purple, Δ*T*_m_ −7.99 °C), AncFT15 (orange, Δ*T*_m_ −16.20 °C), and AncFT16 (yellow, Δ*T*_m_ −15.50 °C). The measurement was performed in triplicates using a sample concentration of ∼1 mg/mL. The standard deviation of the triplicates remained in the range of ±0.01–± 0.05. (B) Fold-increase activity of AncFT variants compared to design template Anc^HLD-RLuc^. Activity was determined spectrophotometrically at 37°, measured in triplicates (see Methods). Error bars correspond to standard deviation in triplicates.

The thermal unfolding and melting temperature (*T*_m_) of protein designs were measured on a nanoDSF (Figure 6A). We detected a single transition step in the unfolding curve of every variant. The results showed that the loop grafting resulted in substantial destabilization of proteins. However, this activity–stability trade-off is a common feature in enzyme engineering.^71^ The grafting of the L9 loop in AncFT9 resulted in a decrease in the *T*_m_ value by ∼8 °C, which is consistent with the previously published data on AncFT with transplanted L9-α4. In that case, the transplanting of the L9-α4 fragment also led to a *T*_m_ value that was 7 °C lower compared to the ancestral variant.^71^ Double-grafting of L9 and L14 loops in AncFT15 and AncFT16 designs caused the *T*_m_ decrease by ∼16 and ∼15.5 °C, respectively (Figure 6A). Subsequently, the luciferase activity of purified proteins was measured, confirming the activity of all of the selected variants (Figure 6B). Precise activity analysis based on the known concentrations of the measured protein revealed a 426-, 408-, and 196-fold increase in luciferase activities in AncFT9, AncFT15, and AncFT16, compared to the original template Anc^HLD-RLuc^ enzyme. Comparatively, the same luciferase activity increase in relation to the original Anc^HLD-RLuc^ enzyme was 148-fold in our previous manual attempt.^33^ This shows the potential of the automated approach presented herein to produce even more successful chimeric variants.

#### Kinetics of the Expressed Variants

The determined steady-state kinetic parameters of luciferase reaction of AncFT 9, 15, and 16, along with previously published^33^ kinetic parameters of Anc^HLD-RLuc^ and AncFT, are summarized in Table 5. All of the predicted chimeric variants showed significantly improved steady-state kinetic parameters of the luciferase reaction over those of initial Anc^HLD-RLuc^. The significant increase in the turnover number *k*_cat_ by almost 3 orders of magnitude and simultaneous improvement of the Michaelis constant *K*_m_ together led to a high improvement in the overall specificity of all constructed FT variants. While AncFT16 resulted in the most improved variant, achieving in all measured parameters values comparable to those measured for our previous rational design AncFT, AncFT9 was the least improved one. Nevertheless, with the exception of *K*_p_, AncFT9 kinetic parameters were comparable or better to those of the reference starting point Anc^HLD-RLuc^.

**Table 5:** Summary of Steady-State Kinetic Parameters Determined for Anc^HLD-RLuc^ and AncFT Variants^a^,^b^

	*K*_m_ [μM]	*k*_cat_ [s^–1^]	*K*_p_ [μM]	kcat/Km [μM^–1^ s^–1^]	K_m_/K_p_
Anc^HLD-RLuc^	1.33 ± 0.001	(3.09 ± 0.01)×10^–4^	0.37 ± 0.01	(2.33 ± 0.01)×10^–4^	3.6 ± 0.1
AncFT	0.06 ± 0.001	0.11 ± 0.001	0.5 ± 0.1	1.73 ± 0.03	0.13 ± 0.03
AncFT9	0.35 ± 0.001	0.13 ± 0.001	0.23 ± 0.001	0.37 ± 0.001	1.54 ± 0.006
AncFT15	0.39 ± 0.004	0.31 ± 0.001	0.34 ± 0.003	0.80 ± 0.005	1.13 ± 0.01
AncFT16	0.06 ± 0.001	0.13 ± 0.001	0.26 ± 0.001	2.28 ± 0.01	0.23 ± 0.002

a *K*_m_ = Michaelis constant; *k*_cat_ = turnover number; *K*_p_ = dissociation constant of the EP complex as a measure of product inhibition; *k*_cat_/*K*_m_ = specificity constant; *K*_m_/*K*_p_ = measure of binding specificity. For *K*_m_ and *K*_m_/*K*_p_, lower is better; for *k*_cat_, *K*_p_, and *k*_cat_/*K*_m_, higher is better.

b Different coelenterazine solutions (4.4 × 0.5^0–7^ μM) were prepared in 100 mM potassium phosphate buffer, pH = 7.5, at 37 °C. Luminescence in the range of 240–720 nanometers was monitored for 1000 s after 10 s of baseline collection or until the luminescence intensity decreased to 0.5% of the maximum value. The final enzyme concentration was either 0.1 or 0.01 μM. All assays were measured in triplicates (standard deviation given as error).

The only parameter that did not show any significant changes was the dissociation constant of EP complex *K*_p_, as a measure of luciferase reaction product inhibition. *K*_p_ stayed on a level comparable to that of the initial Anc^HLD-RLuc^. In the case of AncFT16, a major improvement was obtained in the preference for substrate binding over the reaction product, as measured by the *K*_m_/*K*_p_.

## Discussion and Conclusions

Transferring loops from one protein onto another—loop grafting—has been exploited during the last recent decades as a successful approach in protein engineering.^21,33,42,68^ We recently developed a protocol to automate the task, LoopGrafter^39^ (available at https://loschmidt.chemi.muni.cz/loopgrafter), based on our previous loop grafting experiences.^33,42^ To the best of our knowledge, no other tool has been made available for this specific purpose. The LoopGrafter pipeline was previously shown capable of retrieving the sequences of our previous experiments.^39^ In this work, we showcase that the optimized LoopGrafter pipeline can be effectively used to generate novel viable chimeras that improve the engineered protein activity. Enhancing the catalytic activity of enzymes by modifying their dynamics is a very attractive but challenging protein engineering task.^72,73^ Engaging this challenge, our method aims to transfer dynamic behaviors between the from an active template to a chimera by transplanting loops, expecting that the dynamic changes may impact on the catalytic activity of the chimeras.^41^

Particularly, starting from a bifunctional ancestor encompassing both haloalkane dehalogenase (EC 3.8.5.1) and luciferin 2-monooxygenase (EC 1.13.12.5), the automated transplantation of two particularly dynamic loops (loops 9 and 14) from *Renilla* luciferase increased the bioluminescent activity from purified protein over 400 times. Loop L9 can be identified as a dynamical hinge,^24^ often related to function, while loop L14 emerges as highly cross-correlated to the previous one (Figure 1). The boundaries for transplanting these two regions are automatically explored, and the particular chimeras to be produced are selected by their high scores. Thus, the whole process can be conducted in the absence of previous experimental evidence.

Among all 474 LoopGrafter designs inserting RLuc loops on the bifunctional Anc^HLD-RLuc^ template, we selected 8 of them (2%) based on their puissant stability scores. All of the selected designs (100%) resulted in expressible and soluble proteins. In comparison to initial experimental loop grafting attempts,^21^ where millions of constructs were attempted, this represents a notable improvement in the effectiveness of the process. In a recent study conducted in Kamerlin and Hengge’s laboratories, they aimed to increase the activity of the human protein tyrosine phosphatase PTP1B by replacing the loop WPD with the corresponding loop from the faster *Yersinia pestis* YopH enzyme. They constructed seven single-loop-grafted enzymes and found that four were successfully expressed and soluble and three were also active. This represents a 57% success rate for expression and a 43% success rate for activity.^68^

We are aware that the experimental results presented here for the bifunctional haloalkane dehalogenase/luciferase ancestor system are not generalizable. However, our results suggest that ensuring a good geometrical fitting in the transplanted region and assessing the protein stability across the recombinant sequence space might be critical to generating viable chimeras. Our approach differs from that previously attempted by Kamerlin and co-workers^68^ in two different substantial aspects. First, we started from a reconstructed putative ancestor, which is more stable and more evolvable;^32^ in comparison, the loop grafting on human protein tyrosine phosphatase was done on an already evolutionarily optimized enzyme with substantially higher activity.^68^ Second, our recombination points are limited to the flanking regular secondary structures, whereas the previous study attempted recombination points in the coil part of the loop. However, it needs to be noted that each of these two examples represents a unique system, each with their own intricate protein dynamics driven by complex sequence–dynamics relationships. Thus, generalization to other different systems is far from granted and more case studies would be required to ensure the applicability of LoopGrafter to other enzymes. Besides, our approach is completely automated, may help pinpoint the target loops (Figure 3B, where high correlation between L9 and L14 can be observed, vouching for the inclusion of L14 in the experiments), and does not require prior experimental evidence or proofing (although these are always an advantage).

Furthermore, all selected chimeric variants exhibited increased luminescence activity compared to that of the template Anc^HLD-RLuc^. Loop L9 is known to encompass a particular dynamic behavior responsible for the bioluminescent activity toward luciferin.^41^ Particularly, a wider opening of the cap domain allows the allocation of the substrate in a near-attack conformation close to the catalytic center located inside the core domain of the protein.^41^ By transplanting the loop from its original *Renilla* luciferase scaffold, the dynamic properties were at least partially transferred to the chimeras, and thus, their bioluminescent activity increased. The flexibility predicted by ENMs for the constructed chimeras is in agreement of such alteration of the protein dynamics, suggesting a cross-talk between L9 and L14. The chimeric products of our engineering effort are predicted to have different flexibility and dynamic properties in comparison to those of the template protein Anc^HLD-RLuc^ (Figure S2). Our results contrast with those recently presented by Baker and colleagues on *de novo* design of luciferases^74^ using RosettaDesign^75^ and ProteinMPNN.^76^ While the two tasks are of different difficulty, loop transplantation is easier than *de novo* design, and the results presented in this article support the viability of loop grafting as a protein engineering strategy.^31,66−68^

Various constructs, wherein loops within the Anc^HLD-RLuc^ structure are systematically combined, consistently exhibit elevated catalytic efficiency with the turnover number *k*_cat_ elevated by almost 3 orders of magnitude for all constructed variants. This observation aligns with prior research that establishes a correlation between the catalytic efficiency of different reaction types and the dynamic behavior of proteins, and emphasizes the substantial impact of loops in governing such processes.^77^ The major constraint inherent to these widely used bioluminescent probes is the rapid inactivation of the reaction, resulting in signal instability. Strong product inhibition, delineated by the *K*_m_/*K*_p_ ratio, stands as a primary contributor to the rapid decay observed in the bioluminescence signal following a robust initial flash.^33^ In this aspect, AncFT16 demonstrates a confluence of the utmost achieved reaction specificity with a comparatively minimized burden of product inhibition. The diminished *K*_m_/*K*_p_ ratio of 0.225 ± 0.002 positions AncFT16 at a comparable level to the highly stable glow-type bioluminescent variant AncFT with *K*_m_/*K*_p_ = 0.13 ± 0.03, when contrasted with the flash-type luminescence of RLuc8, exhibiting an order of magnitude higher *K*_m_/*K*_p_ ratio of 1.3 ± 0.1. Therefore, the loop grafting approach presented herein may be leveraged in the future to generate variants that systematically explore and optimize these trade-offs and construct practically useful luciferases for bioimaging, high-throughput screening, diagnostics, therapeutics, and even envisioned light-emitting plants as solution to critical environmental problems associated with our current energy production models.^78^

## Supporting Information Available

Information on the efficient algorithm for transforming a structural superimposition into sequence pairing and figures of SSEs in the Anc^HLD-RLuc^ cap domain and ENM predictions on the flexibility of constructed chimeras (PDF)
Comparison of the LoopGrafter structure-based pairwise sequence alignment to PMLSearch and GTalign (XLSX)
