# Supplementary material for: Automated Engineering Protein Dynamics via Loop Grafting: Improving Renilla Luciferase Catalysis
Source: ACS Catal. 2025 Feb 11;15(4):3391–404. doi: 10.1021/acscatal.4c06207 (PMC11851775; doi:10.1021/acscatal.4c06207)
Supplement: Supplementary file 1 — cs4c06207_si_001.pdf [file cs4c06207_si_001.pdf]

Automated Engineering Protein Dynamics via Loop Grafting: Improving *Renilla* Luciferase Catalysis

Joan Planas-Iglesias[1,2,#], Marika Majerova[1,2,#], Daniel Pluskal[1,2], Michal Vasina[1,2], Jiri Damborsky[1,2], Zbynek Prokop[1,2], Martin Marek[1,2,*], David Bednar[1,2,*]

[1]Loschmidt Laboratories, Department of Experimental Biology and RECETOX, Faculty of Science, Masaryk University, Kotlarska 2, 602 00, Brno, Czech Republic

[2]International Clinical Research Centre, St. Anne's University Hospital, Pekarska 53, 602 00, Brno, Czech Republic

[#]Joint first authors

[*]Corresponding authors: MM: martin.marek@recetox.muni.cz, DB: 222755@mail.muni.cz

Supplementary Materials

Efficient algorithm for transforming a structural superimposition into sequence pairing.

Given two superimposed proteins, *A* and *B*, of different sequence length, *m* and *n*, respectively, their sequences can be represented as ordered sets $A=\{a_1..a_m\}$, $B=\{b_1..b_n\}$ and the Euclidian distances between each of their alpha carbons can be represented in a matrix *D* of dimensions (*m*, *n*). The task of finding the best residue pairing (a_i, b_j) that guarantees optimal local minima consists of an iterative algorithm that finds the minimum of such distances *d*, and removes the paired residues from further consideration. To avoid pairing protein extremes or any otherwise unrelated amino-acid pairs, an additional restriction can be enforced, making the distance *d* between the two paired residues to be lower than a certain threshold *T,* and otherwise leaving the pair (a_i, b_j) unmatched. It has to be noted that optimal local minima are preferred in this case over a global one (such as the one produced, for instance, by the Hungarian method[1]) because the resulting sequence pairing will be guiding the definition of recombination points in between the two input proteins *A* and *B*. It is those recombination points (and not the overall sequence) that are required to be structurally compatible (close in space) in order to produce a successful grafting[2].

The task, as described, involves iterating over the shortest sequence and performing a quadratic search on each iteration. It can be simplified by considering the row and column

ranks of matrix *D*. Let the Euclidian distance values in *D* be denoted $\{d_{11}..d_{ij}..d_{mn}\}$. Every element d_{ij} in *D* can be characterised by its rank on the row *i* and its rank on the column *j* of matrix *D*. Thus, from *D* two matrices *R* and *K*, formed by elements r_{ij} and k_{ij} respectively, and each of dimensions (*m*, *n*) can be derived representing the row and the column ranks, respectively, of every element in *D*. Then, assuming *m* the shortest of the sequences, the same set of parings $\{(a_i,b_j), \ldots\}$ can be obtained by:
iterating from 1 to *m*,

- Let *x* be the iteration number.
- Let L_x be the set formed by any element d_{ij} in *D* that have a rank r_{ij} (row) or k_{ij} (column) equal to *x*
- Pair the residues (a_i, b_j) whose indices correspond to the minimum distance *d* value in the set L_x, provided that the distance *d* is not larger than:
    - the threshold *T*, or
    - the minimum distance in the set L_{x+1} (corresponding to the next iteration)
- Remove any elements from L_x whose indices are i or j (elements encompassing larger distances for already paired positions).

This algorithm speeds up the production of the final sequence pairing by reducing the quadratic search problem to a linear one and by dramatically reducing the number of iterations required. On reasonably well aligned structures, all possible pairs (a_i,b_j) are exhausted during the first iterations or are identified to be separated by a distance *d* larger than the threshold *T*. The threshold used in this work, after manual optimisation on the haloalkane dehalogenase family of proteins, was set to 1.9 Angstrom.

To avoid extreme cases where the N-terminus of one protein superimposes with the C-terminus of the other, a restriction in sequence position difference (sequence offset, s_{ij}) is implemented to prevent such extreme mismatches. In pairs of homologous proteins, pairs of residues typically given a sequence offset. For instance, let the pair (a_i, b_j) a correctly aligned pair in two homologous proteins. Let the *i* index be 1 (representing the first residue in the sequence of protein *A*) and the *j* index be 7 (representing the seventh residue in the sequence of protein *B*). The sequence offset s_{ij} between the two aligned pairs is defined by the

difference in their sequence indices: 7-1 = 6. The next aligned residue pair, would typically be (a_{i+1}, b_{j+1}) == (a_2, b_8), and the s_{ij} will remain as 6 (8-2). To filter out extreme cases of mismatch driven by three-dimensional space proximity, any residue pair that differs in s_{ij} more than 4 times the than the standard deviation of the distribution is unpaired before producing the sequence alignment.

It has to be noted that the obtained sequence pairing does not enforce correlativity of residues in sequence. For instance, a pairing such as $[(a_1, b_1), (a_2, b_5), (a_3, b_4), (a_4, b_3), (a_5, b_2), (a_6, b_6)]$ is possible. This can be also represented as:

```
Sequence A: 123456
Sequence B: 154326
```

Since a requirement of a sequence alignment is that the correlativity of the elements in the sequence is preserved, these elements whose order is reversed in the sequence pairing must result in a gap in order to obtain the final alignment:

```
Sequence A: 12345----6
Sequence B: 1----54326
```

Figure S1

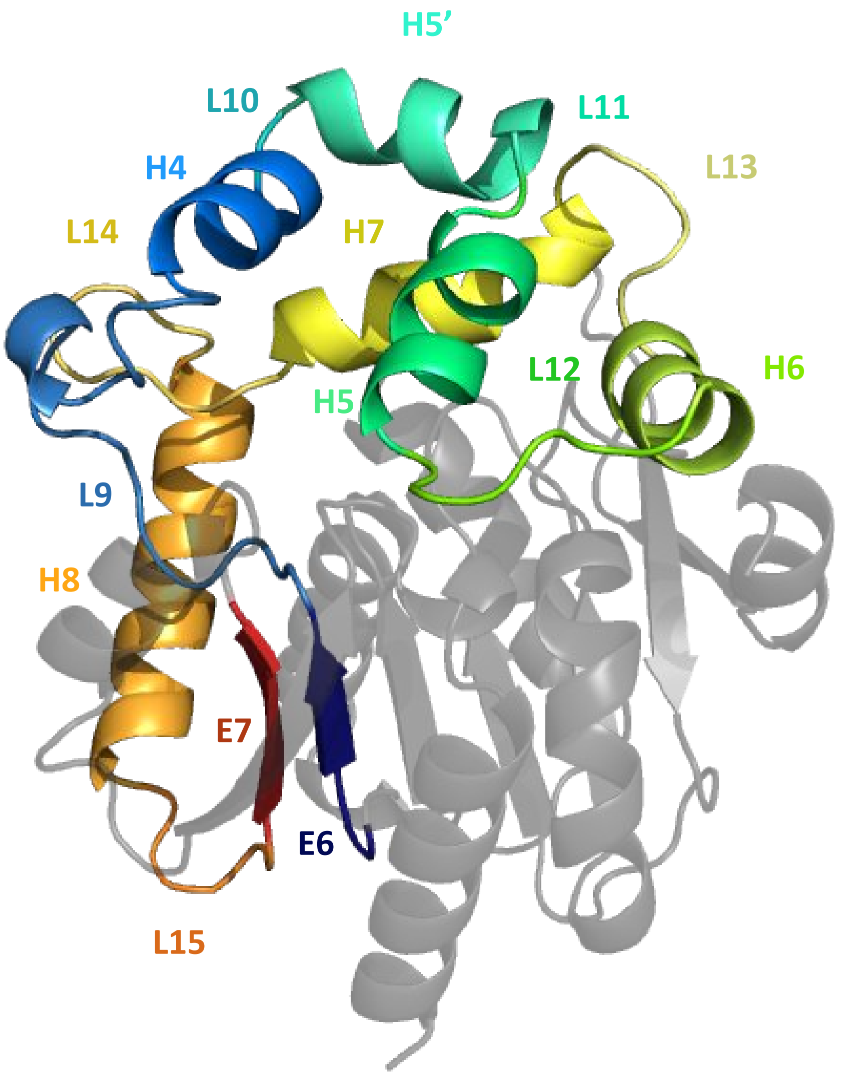

Figure S1: Secondary structure elements in Anc$^{HLD-RLuc}$ cap domain. Individual SSEs are coloured and tagged according to the naming convention used in this work.

Figure S2

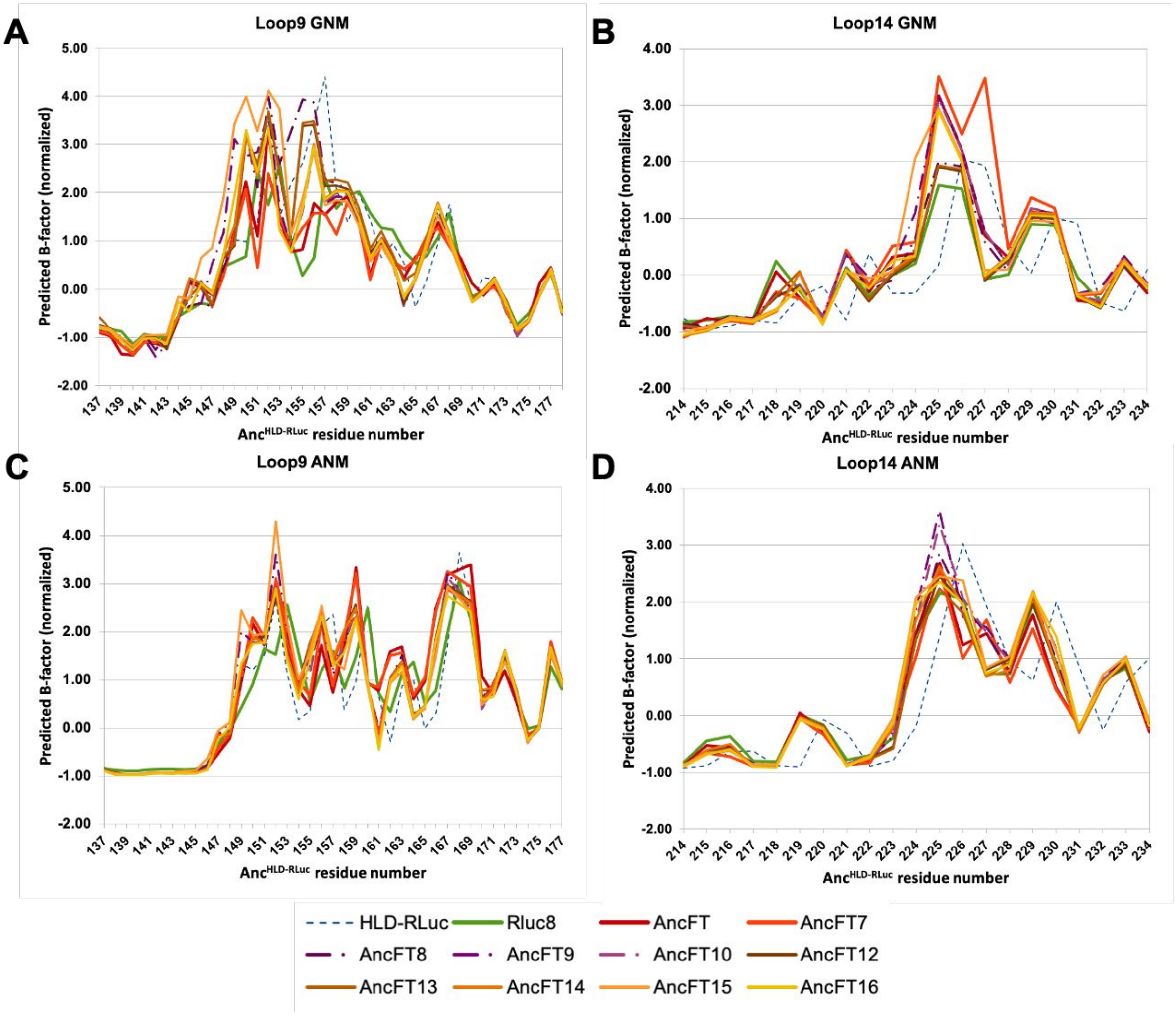

Figure S2: Elastic Network Model predictions on the flexibility of constructed chimeras. Normalized B-factors (y-axis) as a function of the corresponding AncHLD-RLuc residue (x-axis) as calculated by GNM (A, B) and ANM (C, D) on the regions of loop L9 (A, C) and loop L14 (B, D) for different proteins. The template scaffold AncHLD-RLuc (dashed, thin blue line), the insert RLuc8 (solid, thick green line), previously reported chimeras (AncFT and AncFT7, thick solid lines in hues of red) are represented for reference. Chimeras covered in this work only carrying a transplanted L9 from Rluc8 are represented in discontinuous lines and hues of purple. Chimeras covered in this work carrying both L9 and L14 transplanted from RLuc8 are represented in solid lines and hues of orange. It has to be noted that chimeras with only L9 transplanted have L14 profiles different to that of the template AncHLD-RLuc and more similar to RLuc8.

File S1-Sn: Comparison of LoopGrafter structure-based pair-wise sequence alignment to PMLSearch[3] and GTalign[4]. Excel file containing the alignments AncFT7– Anc$^{HLD/RLuc}$ (columns A-E) and AncFT7–RLuc (columns P-T) produced by each of the three methods in different tabs, correspondingly named. Columns A, E, P, and T identify the residue number of the aligned protein. Columns B, D, Q, and S the aligned residues. The row immediately before the first residue identifies the protein name in columns B, D, Q, and S. Before the alignment, scores or other information produced by the alignment method is displayed if available. L9 and L14 regions are highlighted in yellow and orange, respectively.

References

(1) Kuhn, H. W. The Hungarian Method for the Assignment Problem. *Naval Research Logistics Quarterly* **1955**, *2* (1–2), 83–97. https://doi.org/10.1002/nav.3800020109.

(2) Bonet, J.; Segura, J.; Planas-Iglesias, J.; Oliva, B.; Fernandez-Fuentes, N. Frag'r'Us: Knowledge-Based Sampling of Protein Backbone Conformations for de Novo Structure-Based Protein Design. *Bioinformatics* **2014**, *30* (13), 1935–1936. https://doi.org/10.1093/bioinformatics/btu129.

(3) Liu, W.; Wang, Z.; You, R.; Xie, C.; Wei, H.; Xiong, Y.; Yang, J.; Zhu, S. PLMSearch: Protein Language Model Powers Accurate and Fast Sequence Search for Remote Homology. *Nat Commun* **2024**, *15* (1), 2775. https://doi.org/10.1038/s41467-024-46808-5.

(4) Margelevičius, M. GTalign: Spatial Index-Driven Protein Structure Alignment, Superposition, and Search. *Nat Commun* **2024**, *15* (1), 7305. https://doi.org/10.1038/s41467-024-51669-z.
